# Analysis of *in vitro* expression of virulence genes related to antibiotic and disinfectant resistance in *Escherichia coli* as an emerging periodontal pathogen

**DOI:** 10.3389/fcimb.2024.1412007

**Published:** 2024-08-15

**Authors:** Eric Monroy-Pérez, Tania Hernández-Jaimes, Rosario Morales-Espinosa, Gabriela Delgado, Héctor Martínez-Gregorio, Luis Rey García-Cortés, Jennefer Paloma Herrera-Gabriel, Andrea De Lira-Silva, Felipe Vaca-Paniagua, Gloria Luz Paniagua-Contreras

**Affiliations:** ^1^ Facultad de Estudios Superiores Iztacala, Universidad Nacional Autónoma de México, Tlalnepantla, Mexico; ^2^ Departamento de Microbiología and Parasitología, Facultad de Medicina, Universidad Nacional Autónoma de México, Ciudad de México, Mexico; ^3^ Unidad de Biomedicina, Facultad de Estudios Superiores Iztacala, Universidad Nacional Autónoma de México, Tlalnepantla, Mexico; ^4^ Laboratorio Nacional en Salud, Diagnóstico Molecular y Efecto Ambiental en Enfermedades Crónico-Degenerativas, Facultad de Estudios Superiores Iztacala, Universidad Nacional Autónoma de México, Tlalnepantla, Mexico; ^5^ Coordinación de Investigación del Estado de México oriente, Insitituto Mexicano del Seguro Social, Tlalnepantla de Baz, Mexico

**Keywords:** periodontitis, *Escherichia coli*, expression of virulence genes, antibiotic and disinfectant resistance genes, real-time PCR

## Abstract

The collective involvement of virulence markers of *Escherichia coli* as an emerging pathogen associated with periodontitis remains unexplained. This study aimed to implement an *in vitro* model of infection using a human epithelial cell line to determine the virulome expression related to the antibiotic and disinfectant resistance genotype and pulse field gel electrophoresis (PFGE) type in *E. coli* strains isolated from patients with periodontal diseases. We studied 100 strains of *E. coli* isolated from patients with gingivitis (n = 12), moderate periodontitis (n = 59), and chronic periodontitis (n = 29). The identification of *E. coli* and antibiotic and disinfectant resistance genes was performed through PCR. To promote the expression of virulence genes in the strains, an *in vitro* infection model was used in the human epithelial cell line A549. RNA was extracted using the QIAcube robotic equipment and reverse transcription to cDNA was performed using the QuantiTect reverse transcription kit (Qiagen). The determination of virulence gene expression was performed through real-time PCR. Overall, the most frequently expressed adhesion genes among the isolated strains of gingivitis, moderate periodontitis, and chronic periodontitis were *fimH* (48%), *iha* (37%), and *papA* (18%); those for toxins were *usp* (33%); those for iron acquisition were *feoB* (84%), *fyuA* (62%), *irp-2* (61%), and *iroN* (35%); those for protectins were *traT* (50%), *KpsMT* (35%), and *ompT* (28%); and those for pathogenicity islands were *malX* (45%). The most common antibiotic and disinfectant resistance genes among gingivitis, moderate periodontitis, and chronic periodontitis strains were *sul-2* (43%), *bla_SHV_* (47%), *blaTEM* (45%), *tet(A)* (41%), *dfrA1* (32%), *marR-marO* (57%), and *qacEA1* (79%). The findings revealed the existence of a wide distribution of virulome expression profiles related to the antibiotic and disinfectant resistance genotype and PFGE type in periodontal strains of *E. coli.* These findings may contribute toward improving the prevention and treatment measures for periodontal diseases associated with *E. coli.*

## 1 Introduction

Periodontitis is a condition that affects more than 40% of adults in the United States, as well as 11% of the worldwide population, totaling 743 million people (Kwon et al., 2021). This chronic inflammatory disease is caused by the formation of bacterial plaque around the tissues of the teeth that over time causes the formation of periodontal pockets and pathological degradation of the periodontal ligament and alveolar bone, which consequently leads to loss of teeth (Highfield, 2009). The anaerobic bacteria that cause periodontitis include *Prevotella intermedia, Porphyromonas gingivalis, Aggregatibacter actinomycetemcomitans, Fusobacterium nucleatum, Tannerella forsythia*, and *Treponema denticola* (Lopes et al., 2020; Gasmi Benahmed et al., 2022). Recently, *E. coli* has emerged as another pathogen associated with periodontal diseases (da Silva Boghossian et al., 2011). The role and involvement of *E. coli* in the pathogenesis of periodontal disease is unclear; however, the lipopolysaccharides of *Porphyromonas gingivalis* and *E. coli* have been reported to stimulate inflammation in periodontal disease progression (Palaska et al., 2016). In addition to LPS, *E. coli* carries other virulence factors that increase pathogenicity, including genes encoding adhesins, toxins, iron acquisition systems, and protectins (Terlizzi et al., 2017; Bunduki et al., 2021), which are frequently transferred horizontally between strains through pathogenicity islands (PAIs; Desvaux et al., 2020). The frequency and distribution of different markers of *E. coli* virulence have been widely described in uropathogenic (EPEC) and pathogenic cervico-vaginal strains of *E. coli* (CVPEC) (Paniagua-Contreras et al., 2019a; Hyun et al., 2021); however, studies on periodontal *E. coli* strains remain scarce. Our group recently conducted the only study in Mexico on the characterization of the different virulence markers related to phylogroups, serotypes, and phenotype of antibiotic resistance in *E. coli* strains isolated from patients with periodontal disease (Hernández-Jaimes et al., 2022).

The treatment of *E. coli* infections has been complicated in recent years by the emergence of multidrug-resistant strains (Kourtis et al., 2021). Bacterial multidrug-resistance occurs because of the numerous antibiotic resistance genes that code for resistance to streptomycin, aminoglycosides, sulfonamides, chloramphenicol, tetracycline, trimethoprim, quinolones, and beta-lactams through the production of extended-spectrum beta-lactamases (BBLS) (Adamus-Białek et al., 2018; Paniagua-Contreras et al., 2019b; Cox et al., 2022) and also through the chromosomal operon of multiple antibiotic resistance (*marR*; Sharma et al., 2017). Horizontal transfer of antibiotic resistance genes between bacteria through mobile genetic elements such as plasmids, transposons, and integrons has accelerated the spread of multidrug-resistance among bacteria (Zhang et al., 2020). Additionally, integrons often carry genes that result in resistance to disinfectants that are commonly used in hospitals (Hrovat et al., 2023).

The collective involvement of different virulence markers of *E. coli* in periodontal diseases has not been elucidated. Therefore, the purpose of this study was to implement an *in vitro* model of infection using a human epithelial cell line to determine the different expression profiles of virulence genes encoding adhesins (*papA, papG1, papGII*, *papGIII, fimH, afa, sfa, iha, focG*, and *bmaE*), toxins (*cnf-1, hlyA, usp, set-1, astA*, and *tsh*), iron acquisition systems (*iuc, iroN, irp-2, feoB, fyuA*, and *ireA*), protectins (*kpsMT, ompT, iss*, and *traT*), and pathogenicity islands (PAIs; *malX*) related to the genotype of resistance to antibiotics such as streptomycin (*aadA1*), aminoglycosides (*aac(3)-IV)*, sulfonamide (*sul-1, sul-2*, and *sul-3*), beta-lactamases (*bla_SHV_, bla_CITM_, bla_TEM_* and *bla_oxa-1-like_*), chloramphenicol (*cat1* and *cat2*), tetracycline (*tet(A)* and *tet(B)*), trimethoprim *(dfrA1*), quinolones (*qnr* and *flor*), the *marR-marO* locus, and resistance to disinfectants (*qacEA1*) in addition to the expression profiles of genes related to the pulse field gel electrophoresis (PFGE) type in *E. coli* strains isolated from patients with periodontal disease.

## 2 Materials and methods

### 2.1 Patients studied and strain origin

A group of patients (n = 678) with periodontal disease from the dental service of Hospital No. 60 of the Mexican Institute of Social Security (IMSS) located in Mexico state was analyzed. Patients with gingivitis had shown irritation, redness, and inflammation in the gums, while patients with moderate periodontitis had periodontal pockets greater than 4 mm and tooth attachment loss of less than 4 mm; patients with chronic periodontitis had periodontal pockets greater than 5 mm. The study also included a control group of healthy individuals who had no signs and symptoms of periodontal disease (n = 70). The protocol was approved by the institution’s Ethics Committee (Project Identification Code: R-2022-1406-034). All subjects provided informed consent for inclusion prior to participating in the study. The study was conducted in accordance with the Declaration of Helsinki. Patients undergoing periodontal treatment, those with a history of smoking, or those who had received antibiotic treatment in the past six months were excluded from the study. Samples from patients with gingivitis were taken from the surface of the gum using sterile swabs, while samples from patients with moderate and chronic periodontitis were taken with sterile paper tips with the help of periodontal probes. Samples were deposited in brain-heart infusion culture medium (BHI; MCD LAB, Tlalnepantla, Edo. and incubated at 37°C for 24 h. Such samples were then seeded on methylene blue eosin agar (EMB; MCD LAB, Tlalnepantla, Edo.) and incubated at 37° C for 24 h. *E. coli* strains were identified through traditional biochemical testing and amplification of the 16S rRNA gene using polymerase chain reaction (PCR) as previously described (Lane et al., 1985). *E. coli* ATCC 11775 was used as a positive control for PCR assays.

### 2.2 Identification of antibiotic and disinfectant resistance genes

Previously established uniplex PCR primers and conditions were used for the detection of streptomycin resistance genes (*aadA1*), aminoglycosides (*aac(3)-IV*), sulfonamide (*sul-1, sul-2* and *sul-3*), beta-lactamases (*bla_SHV,_ bla_CITM,_ bla_TEM_* y*bla_oxa-1-like_*), chloramphenicol (*cat1* and *cat2*), tetracycline (*tet(A)* and *tet(B)*), trimethoprim *(dfrA1*), quinolones (*qnr* and *flor*), the *marR-marO locus*, and disinfectants (*qacEA1*) (Sáenz et al., 2004; Dallenne et al., 2010; Momtaz et al., 2013). Bacterial DNA was extracted using the boiling method (Ooka et al., 2009), for which four 2-mm diameter colonies of each *E. coli* strain were suspended in 2 mL of sterile deionized water. After boiling for 10 min, the cell suspension was centrifuged at 10,000 x *g* for 5 min. The supernatant was used as template DNA. The final volume per reaction mix for each PCR assay was 20 μL, which contained the following: 1 μL of the first forward and 1 μL of the first reverse (10 pmol, Integrated DNA Technologies, San Diego, CA, USA), 12 μL of Taq DNA Polymerase Master Mix RED (AMPLIQON, Copenhagen, Denmark), 3 μL of nuclease-free water, and 3 μL of DNA template (100 ng). *E. coli* strains Eco1, Eco3, Eco8, Eco52, Eco57, Eco93, Eco115, and Eco182 from cervico-vaginal infections and harboring antibiotic and disinfectant resistance genes were used as positive controls.

### 2.3 Preparation of the human epithelial cell line

To promote the expression of virulence genes in *E. coli* strains, an *in vitro* infection model was implemented as previously described (Cheng et al., 2005; Naglik et al., 2008) using a culture of the A549 epithelial cell line (ATCC CCL-185) from human lung cancer. The A549 cell line was seeded in DMEM medium (Dulbecco’s modified Eagle medium, Corning NY, USA) supplemented with FBS (fetal bovine serum, Corning NY, USA) in a 24-well cell-culture dish and cultured to a confluence of 1.8 × 10^5^ cells/well. All cultures were incubated at 37°C in a 5% CO_2_ atmosphere.

### 2.4 Preparing bacterial dilutions

One colony of each strain of *E. coli* was inoculated in brain-heart infusion broth (MCD Lab, Edo. de México, Mexico) and incubated at 37 °C for 12 h under aerobic conditions. From each *E. coli* culture, 1:4 dilutions were prepared using phosphate-buffered saline to obtain an optical density at 600 nm (OD_600_) of 1.0 using a Beckman DU-7400 spectrophotometer (Laguna Hills, CA, USA). This corresponded to a concentration of 1 × 10^9^ cells/mL. Subsequently, dilutions were performed to obtain a concentration of 2 × 10^6^ cells/mL.

### 2.5 *In vitro* infection of the human epithelial cell line with *E. coli* strains

With the purpose of simulating a periodontal infection, cultures of *E. coli* strains (n=100) from patients with gingivitis, moderate periodontitis and chronic periodontitis were used for infection of the epithelial cell line A546. Volumes of 50 μL were used at a concentration of 2 × 10^6^ cells/mL of each bacterial culture of *E. coli* strains (n=100) to inoculate the surface of each monolayer (1.8 × 10^5^ cells/well) of human epithelial cell culture (A549). The 24-well plates were incubated with 1 mL of F12K (Sigma Aldrich; Merck KGaA, Darmstadt, Germany) and 10% fetal bovine serum at 37°C for 48 h under a 5% CO_2_ atmosphere with saturated humidity. The *E. coli* strain ATCC 11775 was used as a control and bacterial cell adherence to the A549 epithelial cells was verified in the light microscope to ensure bacterial viability during the infection period.

### 2.6 RNA Extraction and reverse transcription to cDNA

Bacterial RNA was extracted using the QIAcube robotic equipment (Qiagen, Hilden, Germany) supplied with the RNeasy (Qiagen) commercial Mini Kit, for which the *E. coli* strains were collected from the surface of the A549 cell line cultures, suspended in 1000 μL of RNA Protect Bacteria (Qiagen) reagent, and centrifuged at 8,000 × g for 10 minutes to obtain the bacterial sediment. In each extraction run, the QIAcube robotic team included a bacterial lysis step with a TE buffer (10 mM Tris-HCl, 1 mM EDTA, pH 8) containing 1 mg/mL lysozyme. The concentration and purity of the total RNA was measured using the NanoDrop 2000 spectrophotometer (Thermo Fisher Scientific, Waltham, MA, USA). The synthesis of the first strand of cDNA was performed using the QuantiTect reverse transcription kit (Qiagen) as per the manufacturer’s instructions.

### 2.7 Determination of virulence gene expression in *E. coli* strains

The expression of virulence genes in *E. coli* strains was determined through real-time PCR using the Rotor-Gene Q 5plex HRM apparatus (Qiagen, Hilden, Germany). In order to establish the expression of *E. coli* virulence genes encoding adhesins (*papA, papG1, papGII*, *papGIII, fimH, afa, sfa, iha, focG*, and *bmaE*), toxins (*cnf-1, hlyA, usp, set-1, astA*, and *tsh*), iron acquisition systems (*iuc, iroN, irp-2, feoB, fyuA*, and *ireA*), protectins (*kpsMT, ompT, iss*, and *traT*), and pathogenicity islands (PAIs; *malX*), previously established primers were used (Rodriguez et al., 2005; Momtaz et al., 2013). A final volume per reaction mix of 20 μL was used for each real-time PCR assay, which consisted of 6 μL of RNase-free water, 10 μL of the QuantiNova™ SYBR Green PCR Kit (Qiagen) master mixture, 1 μL each of forward and reverse primers (1 μM), and 2 μL of cDNA (100 ng). The amplification process comprised an initial activation temperature for 2 min, followed by 40 denaturation cycles at 95°C for 5 s, annealing/extension at 60°C for 10 s. For each quantitative real-time PCR (qPCR) assay, we constructed a standard curve prepared from three cDNA dilutions (100, 200, and 300 ng/μL) of a strain possessing the gene of interest (positive control). From these three cDNA dilutions, the Rotor-Gene Q 5plex HRM System version 2.3.1.49 (Qiagen) software calculated the limit for the definition of the threshold cycle (CT) of each strain. In this way, with the CT values of each strain obtained with respect to the CT of the standard curve of the control strain, the arithmetic mean was obtained and the percentage of expression of each gene was determined. Each real-time PCR assay included a melting curve, two housekeeping genes (*rpoB* and *arcA*), and a non-template control (NTC). Uropathogenic strains of *E. coli* (UPEC) No. 10, 11, 17, 28, 43, 44, 46, 53, 117, and 194 isolated from clinical patients and possessing the virulence genes studied were used as positive controls for the preparation of the standard curve.

### 2.8 Pulsed field gel electrophoresis

The genomic DNA of *E. coli* was prepared using the standardized protocol used for PFGE PulseNet, USA (Ribot et al., 2006). *XbaI* fragments were separated through electrophoresis on 1% agarose gels using 0.5 x TBE buffer at 14°C with an increased pulse time of 2.2 to 54.2 s for 20 h and 6.0 V/cm using the CHEF-MAPPER (Bio-Rad, Hercules, CA, USA). *XbaI* fragment sizes were estimated using *XbaI* fragments from the H9812 global standard of *Salmonella* ser. Braenderup. The images were digitized with the Gel Logic 112 photographic image documentation system (Kodak, Rochester, NY, USA). The BioNumerics v.7.1 software package (Applied Maths, Sint Martens-Latem, Belgium) was used to analyze the fingerprint profile in the PFGE gel. After background subtraction and gel normalization, fingerprints on profiles were typed according to band similarity and dissimilarity, using the Dice similarity coefficient and the arithmetic mean unweighted pairwise clustering method according to the average bond clustering methods. PFGE results were defined according to previously described criteria (Tenover et al., 1995). (1) indistinguishable if their restriction patterns have the same number of bands. (2) Closely related if they present two or three bands of difference. (3) Possibly related if between four and six bands differ. (4) Not related if they differ in seven or more bands.

### 2.9 Unsupervised hierarchical clustering

*E. coli* strains were systematically grouped according to virulome expression, antibiotic resistance genotype and disinfectants, and diagnosis using unsupervised hierarchical clustering based on Euclidean distances for categorical variables. A categorical data matrix that included virulence gene expression, antibiotic-resistance genotype and disinfectants, as well as diagnosis (gingivitis, moderate periodontitis and chronic periodontitis), was created in R (v.3.6.2). The distance of each strain was calculated based on the overall similarity coefficient, which estimates the maximum possible absolute discrepancy between each combined pair of strains. Strains were visualized in a distribution diagram with a dendrogram constructed using ComplexHeatmap (v3.6.2, R core).

### 2.10 Statistical evaluation

Chi-square test was applied using the SPSS statistical software (version 20.0; SPSS Inc., Chicago, IL, USA, A study was performed in the USA) (p < 0.05) to establish the association between the frequency of expression of the virulence genotype of the strains related to the three diagnoses (gingivitis, moderate periodontitis and chronic periodontitis), as well as the association between the frequency of the antibiotic resistance genotype with the three diagnoses, and the association of the frequency of expression of the virulence genotype with the antibiotic resistance genotype in each of the three diagnoses (gingivitis, moderate periodontitis and chronic periodontitis) in the *E. coli* strains.

## 3 Results

### 3.1 Strain origin

Most *E. coli* strains were isolated from patients with moderate periodontitis (59/100; **Table 1**), followed by those with chronic periodontitis (29/100) and gingivitis (12/100). *E. coli* was not identified in the control group of healthy individuals (n=70). After *in vitro* infection of the human epithelial cell line A549, we found that the genes most frequently expressed among the gingivitis, moderate periodontitis, and chronic periodontitis strains were those related to adhesion (*fimH, iha* and *papA*), toxins (*usp* and *hlyA*), iron acquisition (*feoB, fyuA, irp-2* and *iroN*), protectins (*traT, KpsMT* and *ompT*), and pathogenicity islands (*malX*). The frequency of expression of most virulence genes (adhesins, toxins, iron acquisition, protectins and PAIs) was not associated with any of the three diagnoses (gingivitis, moderate periodontitis and chronic periodontitis), however *afa* was significantly associated with gingivitis and *hlyA* with gingivitis and chronic periodontitis (Chi-square test p <0.05, **Table 1**). A higher percentage of expression was also identified for *fimH*, *iha irp-2, fyuA*, and *kpsMT* in strains associated with gingivitis, while the percentages of expression for *usp, iroN*, and *malX* were more prevalent in strains related to moderate periodontitis (**Table 1**).

**Table 1:** Distribution of expression of virulence genes in *E. coli* strains associated with periodontal disease.

Function	Gene	Strain origin (n=100)			p-value	Total (n=100)
		Gingivitis (n=12) No. (%)	Moderate periodontitis (n=59) No. (%)	Chronic periodontitis (n=29) No. (%)		
Adhesins	*papA*	2 (16.6)	10 (16.9)	6 (20.6)	**NS**	18
	*papG1*	0	0	0	–	0
	*papG1I*	0	3 (5)	3 (10.3)	NS	6
	*papGIII*	0	1 (1.6)	0	NS	1
	*fimH*	8 (66.6)	26 (44)	14 (48.2)	NS	48
	*afa*	3 (25)	0	2 (6.8)	**0.0017**	5
	*sfa*	2 (16.6)	5 (8.4)	3 (10.3)	NS	10
	*iha*	7 (58.3)	20 (33.8)	10 (34.4)	NS	37
	*focG*	0	0	0	–	0
	*bmaE*	0	0	0	–	0
Toxins	*cnf-1*	1 (8.3)	4 (6.7)	1 (3.4)	NS	6
	*hlyA*	3 (25)	2 (3.3)	5 (17.2)	**0.0104**	10
	*usp*	3 (25)	21 (35.5)	9 (31)	NS	33
	*set-1*	1 (8.3)	4 (6.7)	2 (6.8)	NS	7
	*astA*	2 (16.6)	3 (5)	2 (6.8)	NS	7
	*tsh*	0	0	0	–	0
Iron acquisition	*iuc*	0	0	0	–	0
	*iroN*	3 (25)	23 (38.9)	9 (31)	NS	35
	*irp-2*	9 (75)	34 (57.6)	18 (62)	NS	61
	*feoB*	11 (91.6)	48 (81.3)	25 (86.2)	NS	84
	*fyuA*	9 (75)	37 (62.7)	16 (55.1)	NS	62
	*ireA*	2 (16.6)	6 (10.1)	1 (3.4)	NS	9
Protectins	*kpsMT*	7 (58.3)	19 (32.2)	9 (31)	NS	35
	*ompT*	4 (33.3)	16 (27.1)	8 (27.5)	NS	28
	*iss*	0	4 (6.7)	0	NS	4
	*traT*	9 (75)	27 (45.7)	14 (48.2)	NS	50
PAIs	*malX*	4 (33.3)	29 (49.1)	12 (41.3)	NS	45

Significant p-values (<0.05) are shown in bold. NS, Not significant.

### 3.2 Frequency of detection of antibiotic and disinfectant resistance genes

The frequency of the antibiotic resistance genotype was not associated with any of the three diagnoses (gingivitis, moderate periodontitis and chronic periodontitis) (Chi-square test p < 0.05, **Table 2**); however, the overall percentages of detection of the genes *aadA1* (streptomycin), *aac(3)-IV* (gentamicin), *sul-2* (sulfonamide), *bla_TEM_* and *bbla_oxa-1-ike_* (beta-lactams), *cat1* (chloramphenicol), *dfrA1* (trimethoprim), and *marR-marO* (locus Mar) were higher in gingivitis-associated strains, while those of *sul-1* (sulfonamide), *bla_SHV_* and *bla_CITM_* (beta-lactam), *tet(A)* and *tet(B)* (tetracycline), *qnr* and *floR* genes (quinolones) were more prevalent in strains related to chronic periodontitis. The frequency of detection of the *qacEA1* (disinfectants) gene was higher in strains associated with moderate and chronic periodontitis (**Table 2**).

**Table 2:** Frequencies of detection of genes encoding resistance to antibiotics and disinfectants in *E. coli* strains associated with periodontal disease.

Strain origin	Number of genes encoding for resistance to antibiotics and disinfectants (%)																	
	Streptomycin	Gentamicin	Sulfonamide			Beta-lactams				Chloramphenicol		Tetracycline		Trimethoprim	Quinolones		Locus MAR	Disinfectant
	*aadA1*	*aac(3)-IV*	*sul-1*	*sul-2*	*sul-3*	*bla_SHV_*	*bla_CITM_*	*bla_TEM_*	*bla_oxa-1-like_*	*cat1*	*cmlA*	*tet(A)*	*tet(B)*	*dfrA1*	*qnr*	*flor*	*marR-marO*	*qacEA1*
Gingivitis (n = 12)	4 (33.3)	4 (33.3)	2 (16.6)	8 (66.6)	3 (25)	3 (25)	1 (8.3)	8 (66.6)	5 (41.6)	5 (41.6)	0 (0)	5 (41.6)	1 (8.3)	5 (41.6)	2 (16.6)	5 (41.6)	8 (66.6)	8 (66.6)
Moderate periodontitis (n = 59)	14 (23.7)	15 (25.4)	17 (28.8)	24 (40.6)	10 (16.9)	26 (44)	8 (13.5)	28 (47.4)	15 (25.4)	11 (18.6)	0 (0)	23 (38.9)	15 (25.4)	18 (30.5)	10 (16.9)	12 (20.3)	33 (55.9)	49 (83)
Chronic periodontitis (n = 29)	8 (27.5)	7 (24.1)	9 (31)	11 (37.9)	7 (24.1)	18 (62)	5 (17.2)	9 (31)	8 (27.5)	2 (6.8)	0 (0)	13 (44.8)	9 (31)	9 (31)	11 (37.9)	13 (44.8)	16 (55.1)	22 (75.8)
p-value	NS	NS	NS	NS	NS	NS	NS	NS	NS	NS	–	NS	NS	NS	NS	NS	NS	NS
Total (n = 100)	26	26	28	43	20	47	14	45	28	18	0	41	25	32	23	30	57	79

NS, Not significant.

### 3.3 Expression of virulence genes related to antibiotic and disinfectant resistance genotype

Our results showed that after infection of the human cell line A549 with *E. coli* strains isolated from gingivitis patients, increased expression of genes associated with adhesion (*fimH* and *iha*), iron acquisition (*irp-2, feoB* and *fyuA*), and protectins (*tratT*) correlated with the presence of genes encoding resistance to sulfonamide (*sul-2*), beta-lactams (*blaTEM*), tetracycline (*tet(A*)), the Mar locus (*marR-marO*), and disinfectants (*qacEA1)* (**Table 3**). Chi-square test analysis revealed that the individual expression of each virulence gene related to the genes encoding resistance to streptomycin (*aadA1*), gentamicin (*aac(3)-IV*), sulfonamide (*sul-2* and *sul-3*), beta-lactams (*bla_SHV_* and *blaTEM*), tetracycline (*tet(A*)), trimethoprim (*dfrA1*), quinolones (*flor*), and disinfectants (*qacEA1) in E. coli* strains from gingivitis was statistically significant (p < 0.05; **Table 3**).

**Table 3:** Frequency of expression of virulence genes in the context of resistance genes to antibiotics and disinfectants in strains of *E. coli* from patients with gingivitis.

Function	Gene	Gingivitis (n = 12) No. (%)																	
		Streptomycin	Gentamicin	Sulfonamide			Beta-lactams				Chloramphenicol		Tetracycline		Trimethoprim	Quinolones		Locus MAR	Disinfectant
		*aadA1*	*aac(3)-IV*	*sul-1*	*sul-2*	*sul-3*	*bla_SHV_*	*bla_CITM_*	*bla_TEM_*	*bla_oxa-1-like_*	*cat1*	*cmlA*	*tet(A)*	*tet(B)*	*dfrA1*	*qnr*	*flor*	*marR- marO*	*qacEA1*
**Adhesins**	*papA*	1 (8.3)	1 (8.3)	0	0	0	0	0	1 (8.3)	0	0	0	1 (8.3)	0	2 (16.6)	0	0	1 (8.3)	1 (8.3)
	*papG1*	0	0	0	0	0	0	0	0	0	0	0	0	0	0	0	0	0	0
	*papG1I*	0	0	0	0	0	0	0	0	0	0	0	0	0	0	0	0	0	0
	*papGIII*	0	0	0	0	0	0	0	0	0	0	0	0	0	0	0	0	0	0
	*fimH*	1 (8.3)	2 (16.6)	2 (16.6)	7 (58.3)	3 (25)	3 (25)	1 (8.3)	4 (33.3)	4 (33.3)	3 (25)	0	4 (33.3)	1 (8.3)	4 (33.3)	2 (16.6)	4 (33.3)	6 (50)	5 (41.6)
	*afa*	1 (8.3)	0	0	2 (16.6)	2 (16.6)	2 (16.6)	1 (8.3)	1 (8.3)	3 (25)	1 (8.3)	0	1 (8.3)	1 (8.3)	1 (8.3)	1 (8.3)	3 (25)	1 (8.3)	2 (16.6)
	*sfa*	0	0	1 (8.3)	2 (16.6)	0	0	0	2 (16.6)	1 (8.3)	0	0	1 (8.3)	0	2 (16.6)	2 (16.6)	1 (8.3)	2 (16.6)	2 (16.6)
	*iha*	2 (16.6)	2 (16.6)	1 (8.3)	6 (50)	2 (16.6)	3 (25)	1 (8.3)	3 (25)	4 (33.3)	3 (25)	0	3 (25)	1 (8.3)	3 (25)	1 (8.3)	2 (16.6)	5 (41.6)	5 (41.6)
	*focG*	0	0	0	0	0	0	0	0	0	0	0	0	0	0	0	0	0	0
	*bmaE*	0	0	0	0	0	0	0	0	0	0	0	0	0	0	0	0	0	0
**Toxins**	*cnf-1*	0	1 (8.3)	1 (8.3)	1 (8.3)	0	0	0	1 (8.3)	0	0	0	1 (8.3)	0	1 (8.3)	1 (8.3)	1 (8.3)	1 (8.3)	1 (8.3)
	*hlyA*	0	1 (8.3)	2 (16.6)	2 (16.6)	0	1 (8.3)	0	1 (8.3)	0	1 (8.3)	0	2 (16.6)	0	2 (16.6)	1 (8.3)	1 (8.3)	2 (16.6)	2 (16.6)
	*usp*	1 (8.3)	1 (8.3)	1 (8.3)	2 (16.6)	1 (8.3)	2 (16.6)	0	0	1 (8.3)	1 (8.3)	0	2 (16.6)	0	1 (8.3)	0	1 (8.3)	1 (8.3)	2 (16.6)
	*set-1*	0	1 (8.3)	1 (8.3)	1 (8.3)	0	0	0	1 (8.3)	0	0	0	1 (8.3)	0	1 (8.3)	1 (8.3)	1 (8.3)	1 (8.3)	1 (8.3)
	*astA*	1 (8.3)	1 (8.3)	0	2 (16.6)	2 (16.6)	1 (8.3)	0	1 (8.3)	1 (8.3)	1 (8.3)	0	2 (16.6)	0	0	0	2 (16.6)	1 (8.3)	1 (8.3)
	*tsh*	0	0	0	0	0	0	0	0	0	0	0	0	0	0	0	0	0	0
**Iron acquisition**	*iuc*	0	0	0	0	0	0	0	0	0	0	0	0	0	0	0	0	0	0
	*iroN*	0	1 (8.3)	2 (16.6)	2 (16.6)	0	1 (8.3)	0	2 (16.6)	1 (8.3)	1 (8.3)	0	1 (8.3)	0	1 (8.3)	2 (16.6)	2 (16.6)	2 (16.6)	3 (25)
	*irp-2*	4 (33.3)	3 (25)	2 (16.6)	6 (50)	2 (16.6)	3 (25)	1 (8.3)	5 (41.6)	3 (25)	4 (33.3)	0	4 (33.3)	1 (8.3)	5 (41.6)	2 (16.6)	3 (25)	6 (50)	6 (50)
	*feoB*	4 (33.3)	5 (41.6)	2 (16.6)	8 (66.6)	3 (25)	3 (25)	1 (8.3)	7 (58.3)	4 (33.3)	5 (41.6)	0	5 (41.6)	1 (8.3)	5 (41.6)	2 (16.6)	4 (33.3)	8 (66.6)	7 (58.3)
	*fyuA*	2 (16.6)	3 (25)	2 (16.6)	8 (66.6)	3 (25)	3 (25)	1 (8.3)	5 (41.6)	4 (33.3)	4 (33.3)	0	5 (41.6)	1 (8.3)	3 (25)	2 (16.6)	4 (33.3)	8 (66.6)	6 (50)
	*ireA*	1 (8.3)	2 (16.6)	1 (8.3)	1 (8.3)	0	1 (8.3)	0	1 (8.3)	0	1 (8.3)	0	0	0	1 (8.3)	0	0	2 (16.6)	2 (16.6)
**Protectins**	*kpsMT*	1 (8.3)	1 (8.3)	3 (25)	6 (50)	2 (16.6)	3 (25)	1 (8.3)	3 (25)	4 (33.3)	2 (16.6)	0	3 (25)	1 (8.3)	4 (33.3)	2 (16.6)	3 (25)	5 (41.6)	5 (41.6)
	*ompT*	1 (8.3)	1 (8.3)	2 (16.6)	4 (33.3)	1 (8.3)	2 (16.6)	0	2 (16.6)	2 (16.6)	1 (8.3)	0	2 (16.6)	0	2 (16.6)	2 (16.6)	2 (16.6)	3 (25)	4 (33.3)
	*iss*	0	0	0	0	0	0	0	0	0	0	0	0	0	0	0	0	0	0
	*traT*	2 (16.6)	3 (25)	2 (16.6)	8 (66.6)	3 (25)	3 (25)	1 (8.3)	5 (41.6)	4 (33.3)	4 (33.3)	0	4 (33.3)	1 (8.3)	4 (33.3)	2 (16.6)	4 (33.3)	7 (58.3)	6 (50)
**PAIs**	*malX*	1 (8.3)	1 (8.3)	2 (16.6)	3 (25)	1 (8.3)	2 (16.6)	0	2 (16.6)	2 (16.6)	1 (8.3)	0	2 (16.6)	0	1 (8.3)	2 (16.6)	3 (25)	2 (16.6)	4 (33.3)
***p-value***		**0.013**	**0.008794**	NS	**2.389E-11**	**0.003996**	**0.002425**	NS	**2.016e-05**	NS	NS	0	**0.003218**	NS	**0.002348**	NS	**0.0001995**	NS	**2.389e-06**

Significant p-values (<0.05) are shown in bold. NS, Not significant.

With respect to the strains of *E. coli* isolated from patients with moderate periodontitis, it was found that the percentages of expression of virulence genes were associated with antibiotic and disinfectant resistance genes (Chi-square test, p < 0.05; **Table 4**). Overall, the highest percentages of gene expression in the strains were detected in the markers of adhesion (*fimH*) and iron acquisition (*irp-2, feoB* and *fyuA*), associated with the presence of genes encoding resistance to beta-lactam (*bla_SHV_* and *bla_TEM_*), the Mar locus (*marR-marO*), and disinfectants (*qacEA1*).

**Table 4:** Frequency of expression of virulence genes related in the context of resistance genes to antibiotics and disinfectants in strains of *E. coli* from patients with moderate periodontitis.

Function	Gene	Moderate periodontitis (n = 59) No. (%)																	
		Streptomycin	Gentamicin	Sulfonamide			Beta-lactams				Chloramphenicol		Tetracycline		Trimethoprim	Quinolones		Locus MAR	Disinfectant
		*aadA1*	*aac(3)-IV*	*sul-1*	*sul-2*	*sul-3*	*bla_SHV_*	*bla_CITM_*	*bla_TEM_*	*bla_oxa-1-like_*	*cat1*	*cmlA*	*tet(A)*	*tet(B)*	*dfrA1*	*qnr*	*flor*	*marR-marO*	*qacEA1*
**Adhesins**	*papA*	1 (1.6)	2 (3.3)	3 (5.0)	6 (10.1)	3 (5.0)	7 (11.8)	0	3 (5.0)	6 (10.1)	0	0	5 (8.4)	2 (3.3)	2 (3.3)	4 (6.7)	2 (3.3)	6 (10.1)	8 (13.5)
	*papG1*	0	0	0	0	0	0	0	0	0	0	0	0	0	0	0	0	0	0
	*papG1I*	0	0	2 (3.3)	3 (5.0)	2 (3.3)	3 (5.0)	0	0	2 (3.3)	0	0	2 (3.3)	0	1 (1.6)	1 (1.6)	1 (1.6)	3 (5.0)	3 (5.0)
	*papGIII*	0	1 (1.6)	0	0	0	0	0	0	1 (1.6)	0	0	1 (1.6)	0	0	1 (1.6)	0	1 (1.6)	1 (1.6)
	*fimH*	6 (10.1)	6 (10.1)	8 (13.5)	8 (13.5)	8 (13.5)	15 (25.4)	4 (6.7)	11 (18.6)	8 (13.5)	3 (5.0)	0	9 (15.2)	6 (10.1)	10 (16.9)	6 (10.1)	8 (13.5)	21 (35.5)	22 (37.2)
	*afa*	0	0	0	0	0	0	0	0	0	0	0	0	0	0	0	0	0	0
	*sfa*	0	0	3 (5.0)	3 (5.0)	0	1	1 (1.6)	4 (6.7)	1 (1.6)	0	0	3 (5.0)	1 (1.6)	1 (1.6)	1 (1.6)	0	3 (5.0)	4 (6.7)
	*iha*	6 (10.1)	9 (15.2)	5 (8.4)	6 (10.1)	5 (8.4)	10 (16.9)	3 (5.0)	9 (15.2)	8 (13.5)	7 (11.8)	0	7 (11.8)	6 (10.1)	6 (10.1)	6 (10.1)	5 (8.4)	11 (18.6)	16 (27.1)
	*focG*	0	0	0	0	0	0	0	0	0	0	0	0	0	0	0	0	0	0
	*bmaE*	0	0	0	0	0	0	0	0	0	0	0	0	0	0	0	0	0	0
**Toxins**	*cnf-1*	0	1 (1.6)	0	1 (1.6)	0	0	1 (1.6)	3 (5.0)	0	0	0	0	1 (1.6)	1 (1.6)	1 (1.6)	1 (1.6)	4 (6.7)	3 (5.0)
	*hlyA*	0	0	2 (3.3)	1 (1.6)	0	2 (3.3)	0	0	0	0	0	2 (3.3)	0	0	0	0	2 (3.3)	2 (3.3)
	*usp*	4 (6.7)	8 (13.5)	6 (10.1)	6 (10.1)	6 (10.1)	13 (22.0)	4 (6.7)	8 (13.5)	7 (11.8)	3 (5.0)	0	6 (10.1)	4 (6.7)	6 (10.1)	5 (8.4)	7 (11.8)	15 (25.4)	20 (33.8)
	*set-1*	1 (1.6)	1 (1.6)	1 (1.6)	2 (3.3)	1 (1.6)	1 (1.6)	2 (3.3)	3 (5.0)	1 (1.6)	1 (1.6)	0	0	1 (1.6)	1 (1.6)	1 (1.6)	1 (1.6)	4 (6.7)	4 (6.7)
	*astA*	1 (1.6)	0	1 (1.6)	0	0	2 (3.3)	2 (3.3)	1 (1.6)	2 (3.3)	1 (1.6)	0	0	2 (3.3)	1 (1.6)	0	0	3 (5.0)	3
	*tsh*	0	0	0	0	0	0	0	0	0	0	0	0	0	0	0	0	0	0
**Iron acquisition**	*iuc*	0	0	0	0	0	0	0	0	0	0	0	0	0	0	0	0	0	0
	*iroN*	4 (6.7)	5 (8.4)	6 (10.1)	14 (23.7)	3 (5.0)	8 (13.5)	2 (3.3)	14 (23.7)	5 (8.4)	6 (10.1)	0	10 (16.9)	7 (11.8)	7 (11.8)	4 (6.7)	3 (5.0)	10 (16.9)	19 (32.2)
	*irp-2*	11 (18.6)	10 (16.9)	10 (16.9)	14 (23.7)	7 (11.8)	16 (27.1)	8 (13.5)	17 (28.8)	11 (18.6)	5 (8.4)	0	15 (25.4)	9 (15.2)	12 (20.3)	6 (10.1)	7 (11.8)	19 (32.2)	27 (45.7)
	*feoB*	13 (22.0)	12 (20.3)	13 (22.0)	19 (32.2)	9 (15.2)	23 (38.9)	5 (8.4)	23 (38.9)	14 (23.7)	11 (18.6)	0	19 (32.2)	14 (23.7)	15 (25.4)	8 (13.5)	10 (16.9)	28 (47.4)	39 (66.1)
	*fyuA*	11 (18.6)	11 (18.6)	10 (16.9)	9 (15.2)	9 (15.2)	20 (33.8)	6 (10.1)	14 (23.7)	10 (16.9)	5 (8.4)	0	14 (23.7)	8 (13.5)	13 (22.0)	7 (11.8)	10 (16.9)	29 (49.1)	33 (55.9)
	*ireA*	1 (1.6)	3 (5.0)	0	2 (3.3)	2 (3.3)	4 (6.7)	1 (1.6)	2 (3.3)	2 (3.3)	1 (1.6)	0	2 (3.3)	2 (3.3)	2 (3.3)	3 (5.0)	0	5 (8.4)	6 (10.1)
**Protectins**	*kpsMT*	6 (10.1)	7 (11.8)	6 (10.1)	7 (11.8)	3 (5.0)	10 (16.9)	5 (8.4)	9 (15.2)	8 (13.5)	4 (6.7)	0	6 (10.1)	6 (10.1)	6 (10.1)	6 (10.1)	5 (8.4)	14 (23.7)	16 (27.1)
	*ompT*	4 (6.7)	5 (8.4)	4 (6.7)	6 (10.1)	6 (10.1)	8 (13.5)	4 (6.7)	8 (13.5)	6 (10.1)	3 (5.0)	0	6 (10.1)	4 (6.7)	7 (11.8)	4 (6.7)	3 (5.0)	12 (20.3)	15 (25.4)
	*iss*	1 (1.6)	2 (3.3)	1 (1.6)	0	0	1 (1.6)	0	2 (3.3)	1 (1.6)	2 (3.3)	0	2 (3.3)	2 (3.3)	0	0	1 (1.6)	2 (3.3)	3 (5.0)
	*traT*	6 (10.1)	6 (10.1)	8 (13.5)	9 (15.2)	5 (8.4)	17 (28.8)	5 (8.4)	10 (16.9)	11 (18.6)	4 (6.7)	0	12 (20.3)	8 (13.5)	7 (11.8)	5 (8.4)	8 (13.5)	20 (33.8)	23 (38.9)
**PAIs**	*malX*	5 (8.4)	9 (15.2)	7 (11.8)	11 (18.6)	4 (6.7)	15 (25.4)	4 (6.7)	12 (20.3)	8 (13.5)	5 (8.4)	0	7 (11.8)	7 (11.8)	9 (15.2)	7 (11.8)	7 (11.8)	21 (35.5)	25 (42.3)
***p-value***		**0.0004**	**0.0004**	**0.0004**	**0.0004**	**0.0004**	**0.0004**	**0.0004**	**0.0004**	**0.0004**	**0.0004**	**-**	**0.0004**	**0.0004**	**0.0004**	**0.0004**	**0.0004**	**0.0004**	**0.0004**

Significant p-values (<0.05) are shown in bold. NS, Not significant.

The percentages of expression of virulence genes were associated with the majority of antibiotic and disinfectant resistance genes in *E. coli* strains isolated from patients with chronic periodontitis, except in *cat1*, which was non-significant (Chi-square test, p < 0.05; **Table 5**). The highest percentages of expression were detected for the genes associated with adhesion (*fimH* and *iha*), acquisition of iron (*irp-2, feoB* and *fyuA*), and protectins (*tratT*), as well as pathogenicity islands (PAIs; *malX*), which were associated with the presence of genes related to resistance to beta-lactam (*bla_SHV_*), the Mar locus (*marR-marO*), and disinfectants (*qacEA1*).

**Table 5:** Frequency of expression of virulence genes related in the context of resistance genes to antibiotics and disinfectants in *E. coli* strains from patients with chronic periodontitis.

Function	Gene	Chronic periodontitis (n = 29) No. (%)																	
		Streptomycin	Gentamicin	Sulfonamide			Beta-lactams				Chloramphenicol		Tetracycline		Trimethoprim	Quinolones		Locus MAR	Disinfectant
		*aadA1*	*aac(3)-IV*	*sul-1*	*sul-2*	*sul-3*	*bla_SHV_*	*bla_CITM_*	*bla_TEM_*	*bla_oxa-1-like_*	*cat1*	*cmlA*	*tet(A)*	*tet(B)*	*dfrA1*	*qnr*	*flor*	*marR-marO*	*qacEA1*
**Adhesins**	*papA*	0	3 (10.3)	1 (3.4)	3 (10.3)	3 (10.3)	5 (17.2)	2 (6.8)	0	3 (10.3)	0	0	2 (6.8)	2 (6.8)	3 (10.3)	3 (10.3)	4 (13.7)	5 (17.5)	6 (20.6)
	*papG1*	0	0	0	0	0	0	0	0	0	0	0	0	0	0	0	0	0	0
	*papG1I*	0	2 (6.8)	1 (3.4)	2 (6.8)	2 (6.8)	3 (10.3)	1 (3.4)	0	2 (6.8)	0	0	1 (3.4)	1 (3.4)	0	0	2 (6.8)	2 (6.8)	3 (10.3)
	*papGIII*	0	0	0	0	0	0	0	0	0	0	0	0	0	5 (17.2)	0	0	0	0
	*fimH*	2 (6.8)	5 (17.2)	6 (20.6)	6 (20.6)	4 (13.7)	10 (34.4)	3 (10.3)	2 (6.8)	5 (17.2)	1 (3.4)	0	7 (24.1)	5 (17.2)	1 (3.4)	6 (20.6)	8 (27.5)	12 (41.3)	13 (44.8)
	*afa*	0	1 (3.4)	1 (3.4)	1 (3.4)	1 (3.4)	2 (6.8)	0	0	2 (6.8)	0	0	1 (3.4)	1 (3.4)	1 (3.4)	0	0	1 (3.4)	2 (6.8)
	*sfa*	0	1 (3.4)	0	1 (3.4)	1 (3.4)	2 (6.8)	1 (3.4)	1 (3.4)	2 (6.8)	0	0	1 (3.4)	1 (3.4)	1 (3.4)	2 (6.8)	3 (10.3)	3 (10.3)	3 (10.3)
	*iha*	0	3 (10.3)	5 (17.2)	7 (24.1)	5 (17.2)	9 (31.0)	3 (10.3)	1 (3.4)	6 (20.6)	0	0	4 (13.7)	4 (13.7)	5 (17.2)	4 (13.7)	7 (24.1)	8 (27.5)	9 (31.0)
	*focG*	0	0	0	0	0	0	0	0	0	0	0	0	0	0	0	0	0	0
	*bmaE*	0	0	0	0	0	0	0	0	0	0	0	0	0	0	0	0	0	0
**Toxins**	*cnf-1*	0	0	0	1 (3.4)	1 (3.4)	1 (3.4)	1 (3.4)	0	1 (3.4)	0	0	1 (3.4)	0	1 (3.4)	0	1 (3.4)	1 (3.4)	1 (3.4)
	*hlyA*	0	2 (6.8)	2 (6.8)	4 (13.7)	4 (13.7)	5 (17.2)	2 (6.8)	0	4 (13.7)	0	0	2 (6.8)	3 (10.3)	3 (10.3)	2 (6.8)	4 (13.7)	4 (13.7)	5 (17.2)
	*usp*	0	3 (10.3)	4 (13.7)	5 (17.2)	3 (10.3)	8	3 (10.3)	1 (3.4)	5 (17.2)	0	0	4 (13.7)	5 (17.2)	4 (13.7)	5 (17.2)	6 (20.6)	8 (27.5)	8 (27.5)
	*set-1*	0	0	1 (3.4)	1 (3.4)	1 (3.4)	1 (3.4)	1 (3.4)	1 (3.4)	1 (3.4)	0	0	1 (3.4)	0	2 (6.8)	0	2 (6.8)	1 (3.4)	2 (6.8)
	*astA*	2 (6.8)	0	0	0	0	1 (3.4)	0	1 (3.4)	0	1 (3.4)	0	1 (3.4)	1 (3.4)	1 (3.4)	1 (3.4)	0	1 (3.4)	1 (3.4)
	*tsh*	0	0	0	0	0	0	0	0	0	0	0	0	0	0	0	0	0	0
**Iron acquisition**	*iuc*	0	0	0	0	0	0	0	0	0	0	0	0	0	0	0	0	0	0
	*iroN*	3 (10.3)	4 (13.7)	3 (10.3)	4 (13.7)	3 (10.3)	5 (17.2)	2 (6.8)	4 (13.7)	3 (10.3)	0	0	3 (10.3)	3 (10.3)	4 (13.7)	2 (6.8)	7 (24.1)	6 (20.6)	9 (31.0)
	*irp-2*	8 (27.5)	5 (17.2)	6 (20.6)	8 (27.5)	7 (24.1)	13 (44.8)	4 (13.7)	5 (17.2)	8 (27.5)	0	0	7 (24.1)	7 (24.1)	6 (20.6)	6 (20.6)	11 (37.9)	12 (41.3)	15 (51.7)
	*feoB*	8 (27.5)	5 (17.2)	7 (24.1)	9 (31.0)	7 (24.1)	15 (51.7)	4 (13.7)	8 (27.5)	8 (27.5)	2 (6.8)	0	12 (41.3)	8 (27.5)	8 (27.5)	10 (34.4)	11 (37.9)	14 (48.2)	21 (72.4)
	*fyuA*	3 (10.3)	5 (17.2)	7 (24.1)	8 (27.5)	7 (24.1)	12 (41.3)	3 (10.3)	3 (10.3)	8 (27.5)	0	0	7 (24.1)	5 (17.2)	6 (20.6)	5 (17.2)	9 (31.0)	10 (34.4)	14 (48.2)
	*ireA*	0	0	1 (3.4)	0	0	1 (3.4)	0	0	0	0	0	1 (3.4)	1 (3.4)	0	1 (3.4)	0	1 (3.4)	1 (3.4)
**Protectins**	*kpsMT*	0	2 (6.8)	4 (13.7)	5 (17.2)	5 (17.2)	8 (27.5)	3 (10.3)	1 (3.4)	6 (20.6)	0	0	4 (13.7)	3 (10.3)	5 (17.2)	4 (13.7)	6 (20.6)	7 (24.1)	8 (27.5)
	*ompT*	1 (3.4)	3 (10.3)	4 (13.7)	5 (17.2)	4 (13.7)	7 (24.1)	1 (3.4)	1 (3.4)	5 (17.2)	0	0	4 (13.7)	3 (10.3)	3 (10.3)	1 (3.4)	5 (17.2)	6 (20.6)	8 (27.5)
	*iss*	0	0	0	0	0	0	0	0	0	0	0	0	0	0	0	0	0	0
	*traT*	2 (6.8)	5 (17.2)	7 (24.1)	6 (20.6)	6 (20.6)	11 (37.9)	4 (13.7)	3 (10.3)	7 (24.1)	0	0	6 (20.6)	5 (17.2)	6 (20.6)	5 (17.2)	10 (34.4)	11 (37.9)	13 (44.8)
**PAIs**	*malX*	1 (3.4)	3 (10.3)	3 (10.3)	6 (20.6)	5 (17.2)	11 (37.9)	3 (10.3)	1 (3.4)	7 (24.1)	0	0	3 (10.3)	4 (13.7)	5 (17.2)	4 (13.7)	6 (20.6)	9 (31.0)	11 (37.9)
***p-value***		**0.0004**	**0.0004**	**0.0004**	**0.0004**	**0.0004**	**0.0004**	**0.01249**	**0.0004**	**0.0004**	NS	–	**0.0004**	**0.0004**	**0.0004**	**0.0004**	**0.0004**	**0.0004**	**0.0004**

Significant p-values (<0.05) are shown in bold. NS, Not significant.

### 3.4 Pulsed field gel electrophoresis and virulence genotype expression associated with antibiotic and disinfectant resistance

**Figure 1:**
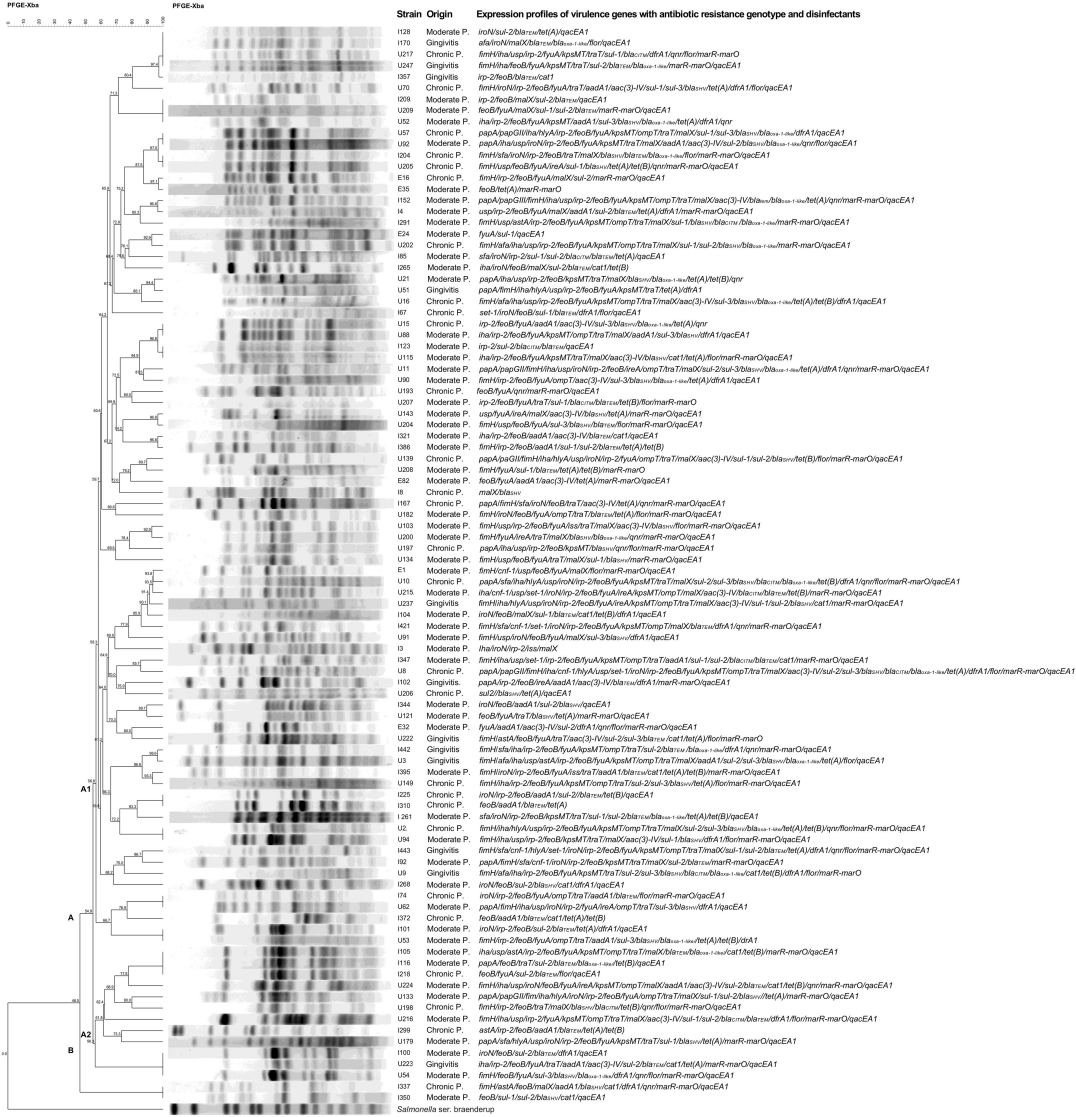
Dendrogram of the pulsed field gel electrophoresis (PFGE) profile and expression patterns of virulence genes and genotype of resistance to antibiotics and disinfectants in *E*. *coli* strains isolated from patients with periodontal disease. The dendrogram was constructed using the Dice similarity coefficient and the unweighted pairwise clustering method with arithmetic mean clustering methods using PFGE images of *XbaI*-digested genomic DNA. The scale bar shows the correlation coefficient (%). The capital letters **(A, B)** represent the two main clades produced. The letters A1 and A2 represent subgroups of strains. Strain number, origin, and expression pattern of virulence genes associated with the antibiotic and disinfectant resistance genotype are shown on the right.

Our findings demonstrated that the *Xba1* fragments of the strains analyzed using PFGE were distributed in two main clades (A and B; **Figure 1**). Clade A comprised 94 strains distributed in two subgroups, A1 (n = 82) and A2 (n = 12). The A1 subgroup showed a Dice similarity index > 54.9% between the strains, and the A2 showed a Dice similarity index of 56.3%. Subgroup A1 comprised 10 different PFtypes (pulse field types). Among these, a specific PFtype composed of strains l128, l170, U217, and U247 from different origins (gingivitis, moderate periodontitis, and chronic periodontitis) showed diverse expression patterns of virulence genes associated with the genotype of resistance to antibiotics and disinfectants. Within this PFtype, the U217 strain had the broadest pattern (*fimH/iha/usp/irp-2/fyuA/KpsMT/traT/sul-1/blaCITM/dfrA1/qnr/flor/marR-marO*). In subgroup A1, another PFtype was also found, consisting of three strains from patients with moderate periodontitis (l209, U209, and U52), and PFtypes composed of paired strains (U57 and U92; l204 and U205); all of these PFtypes exhibited different expression patterns of virulence genes associated with the antibiotic and disinfectant resistance genotype.

Similarly, in subgroup A2, it was found that the PFtype, composed of three strains (l105, l116, and l218) of different origin, showed different expression patterns of virulence genes associated with the genotype of resistance to antibiotics and disinfectants.

Clade B contained a single PFtype composed of strains l337 (chronic periodontitis) and l350 (moderate periodontitis), both with different expression patterns of virulence genes associated with the antibiotic and disinfectant resistance genotype.

PFGE analysis was only performed on 96 *E. coli* strains, as cutting of the chromosomal DNA of U1, l240 and U93 (moderate periodontitis), and E37 (chronic periodontitis) strains with the *Xba1* enzyme was not possible.

The overall results found are outstanding and showed a wide diversity of the PFtype in the periodontal strains analyzed, where some of these PFtype presented strains identical to each other, but with different clinical origin and expression profile of the virulence genotype associated with the genotype of resistance to antibiotics and disinfectants.

**Figure 2:**
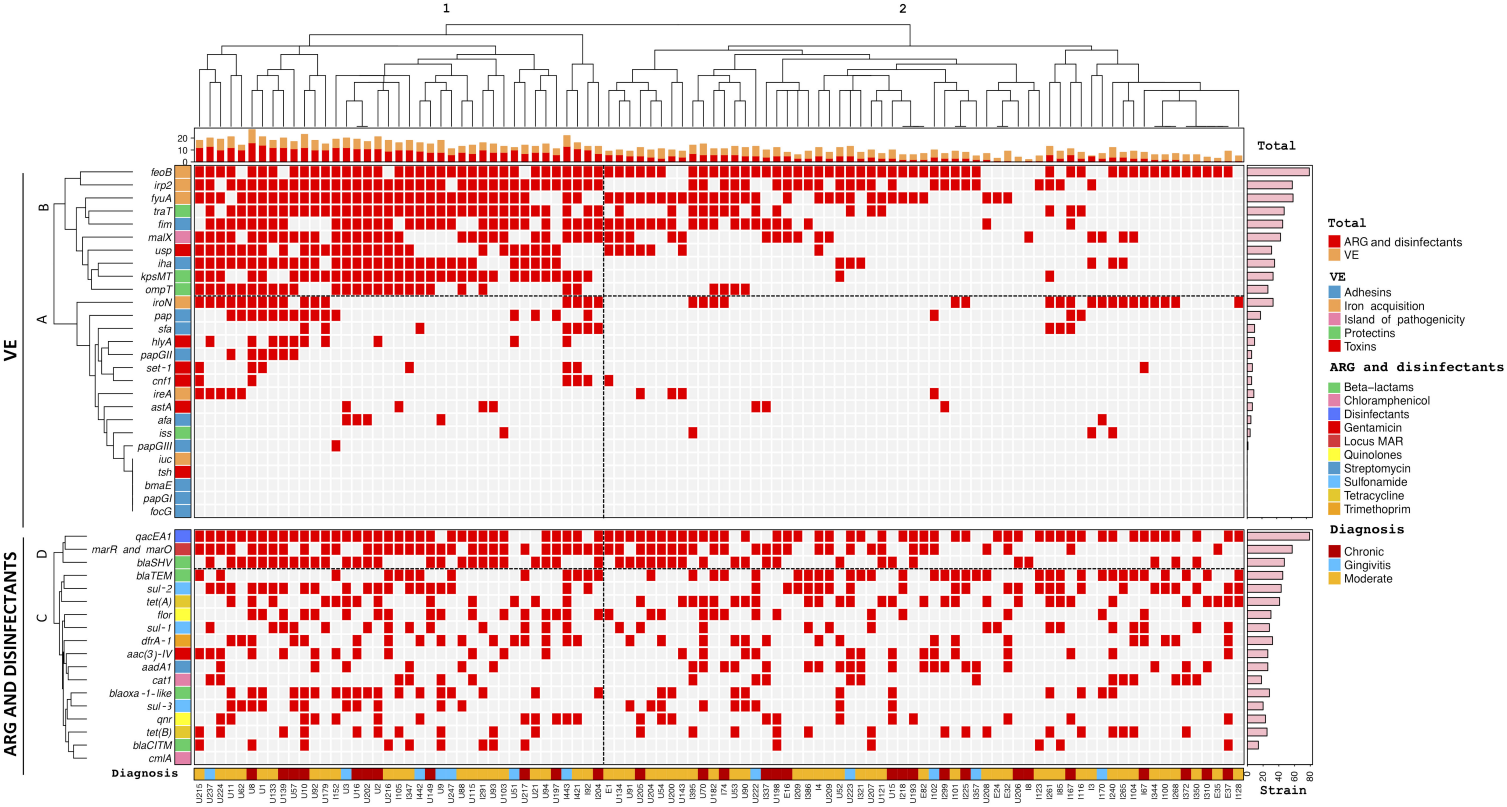
Hierarchical clustering of *Escherichia coli* strains according to virulome expression (VE) related to antibiotic resistance genotype (ARG) and disinfectants and diagnosis. Positivity and negativity for a particular genotype is represented by a red and gray rectangle, respectively. Upper panel: Virulome expression (VE). Lower panel: Antibiotic resistance genotype (ARG) and disinfectants. Left axis: Expression of virulence factors (clades A and B) and detected resistance genes to antibiotics and disinfectants (clades C, D). Right axis: Absolute frequency of expression of virulence genes and antibiotic resistance genes. Top: Cladogram total of VE and ARG and disinfectants of the strains. Bottom: diagnosis nd origin of the strains.

Unsupervised hierarchical clustering analysis showed two large groups based on similarities between *E. coli* strains, based on virulome expression and its relationship with antibiotic and disinfectant resistance genotype, and diagnosis (**Figure 2**). Group 1 was distributed into several subgroups, consisted of 39 strains (U215 to I204) from the three diagnoses (gingivitis, moderate P. and chronic P.), while group 2 consisted of 61 strains (E1 to I128), also the three diagnoses. The cladogram of the virulome expression showed groups A and B and the cladogram of the genotype of resistance to antibiotics and disinfectants showed groups C and D. Overall, the strains of group 1 showed a high frequency of expression of 7 genes (*feoB, irp2, fyuA, traT, fim, malX, usp, iha, kpsMT* and *ompT*) of the viruloma (group B) with respect to the strains of group 2. The strains of group 1 and group 2 showed a high frequency of the genes *qacEA1, mar and marO* and *bla_SHV_* within the group D cladogram of the antibiotic and disinfectant resistance genotype.

## 4 Discussion

Periodontitis is a multifactorial, chronic, inflammatory disease that affects a large part of the world’s population (Liu et al., 2023). In 2019 alone, one billion one hundred million people worldwide were diagnosed with periodontitis, mainly in less developed countries (Chen et al., 2021). In our study, we analyzed a group of *E. coli* strains isolated from patients with periodontal disease (100/678), mainly from patients with moderate periodontitis (59/100) and chronic periodontitis (29/100). The *E. coli* species was not detected in any of the healthy individuals in our control group. In Mexico, the prevalence of periodontitis reported among the indigenous population of the Chiapas state was 8.7% (García-Pérez et al., 2016), while in the USA, the incidence of periodontitis has been reported in 42% of the population, categorized into severe periodontitis (7.8%) and non-severe periodontitis (34.4%) (Eke et al., 2020).

Our team previously conducted molecular characterization of these same strains of *E. coli* (n = 100) as an emerging pathogen associated with periodontal disease, broadly correlating the frequency of the virulence genotype with phylogroups, serotypes, biofilm formation, and antibiotic resistance phenotype (Hernández-Jaimes et al., 2022); however, there are currently no studies on the expression of virulence markers in periodontal strains of *E. coli*. Therefore, with the same strains of *E. coli* (n = 100), we used an *in vitro* infection model in the A549 human epithelial cell line to determine the different expression profiles of the virulence genotype related to the antibiotic and disinfectant resistance genotype and its association with the pulse field gel electrophoresis (PFGE) type. The overall results of this study demonstrated that the percentages of expression of the different virulence genes in the strains associated with gingivitis, moderate periodontitis, and chronic periodontitis after infection of the A549 line were very similar (**Table 1**), except for the expression of the *papA* gene, where statistically significant differences were found (p < 0.05). The adhesion genes *fimH* and *iha* were more frequently expressed in strains from patients with gingivitis than in those from patients with moderate periodontitis and chronic periodontitis. *papA* was expressed more frequently in strains associated with chronic periodontitis and, *sfa* was expressed more frequently in strains isolated from gingivitis patients. The expression of these adhesion markers could increase the persistence and chronicity of periodontal infections, since it has been reported that the FimH protein is responsible for the formation, proliferation, invasion, and internalization of biofilms, favoring the formation of intracellular bacterial communities (IBCs) (Zhou et al., 2001; Lewis et al., 2016). The Iha protein adheres to epithelial cells in urinary tract infections (Johnson et al., 2005), and the PapA protein is associated with pyelonephritis in acute infections caused by uropathogenic *E. coli* (Lavigne et al., 2016), while the sfa protein is responsible for binding to epithelial cells and facilitating bacterial spread within host tissues, causing bacteremia, meningitis, and ascending urinary tract infections (Bien et al., 2012). Expression of adhesion genes in periodontal *E. coli* strains *in vivo* or *in vitro* has not been studied; however, we previously described higher expression percentages for *fimH* (58.2%)*, iha* (64.9%)*, papGII* (15.4%)*, papGIII* (5.7%), and *afa* in UPEC strains (12.4%) (Paniagua-Contreras et al., 2017).

Bacterial toxins cause inflammation, tissue damage, and degradation of the host’s defenses, favoring bacterial penetration and nutrient acquisition (Whelan et al., 2023). In this study, genes encoding toxins such as *usp* were found to be expressed more frequently in *E. coli* strains associated with moderate periodontitis and chronic periodontitis; *hlyA* was expressed more in *E. coli* strains associated with gingivitis and chronic periodontitis, and *astA* and *cnf-1* had the highest expression values in strains isolated from patients with gingivitis. We previously reported higher percentages of expression for *usp* (68%)*, set-1* (31.4%), and *astA* (30.9%) in UPEC strains (n = 194) (Paniagua-Contreras et al., 2017), while the expression percentage for *hlyA* was very similar between periodontal (10%) and UPEC strains (11.8%). Our findings regarding expression of toxin genes in periodontal strains of *E. coli* during the *in vitro* model of infection of the human epithelial cell line A549 revealed that during the pathogenesis of periodontal disease in patients, there could be collective co-expression of toxin genes, which would favor the chronicity of infections. Hemolysin A (HlyA) smooths red blood cells, causes damage to the endothelium, and induces cellular apoptosis of neutrophils, lymphocytes, and renal cells (Jonas et al., 1993; Russo et al., 2005), while the specific uropathogenic protein (Usp) damages epithelial tissue and is expressed in strains that cause pyelonephritis, prostatitis, and bacteremia of urinary origin (Nipič et al., 2013). Cytotoxic necrosis factor 1 (CNF-1) interferes with polymorphonuclear phagocytosis, causes apoptotic death of bladder epithelial cells and contributes to meningitis (Mills et al., 2000).

In this work, we found that in the strains associated with gingivitis, the genes encoding iron uptake systems most frequently expressed after infection of the A549 cell line were *feoB, fyuA*, and *irp-2*, while the percentage of *iroN* expression was higher in strains associated with moderate periodontitis. The high frequency of simultaneous expression of genes encoding iron capitation systems shows the ability of the strains to persist, multiply, and cause persistent periodontal infections in patients, because iron is a bacterial element necessary for the DNA synthesis, electron transport, and peroxide metabolism (Gonciarz and Renslo, 2021). The expression of the *feoB* and *fyuA* genes has been frequently reported in uropathogenic strains causing urinary tract infections (Ali et al., 2019). The percentages of global expression of *irp-2* (61%) and *iroN* (35%) detected in *E. coli* strains isolated from patients with periodontal disease are very similar to those described by our group working on cervico-vaginal pathogenic *E. coli* strains (n = 200), where the prevalence of *irp-2* was 58.5% and that of *iroN* was 32.5% (Monroy-Pérez et al., 2020).

The distribution of the expression of the protectin genes *traT, kpsMT*, and *ompT* was very similar among the strains associated with moderate periodontitis and chronic periodontitis; however, the percentage of *kpsMT* expression was higher in the strains isolated from patients with gingivitis. The high expression of protectin genes in periodontal *E. coli* strains indicates the ability of the strains to cause chronic periodontal infections, because the KpsMT capsular antigen protects the bacterium against phagocytosis, opsonization, and lysis (Pompilio et al., 2018); OmpT degrades host defensins (Hui et al., 2010); and TraT confers resistance to serum (Daga et al., 2019). The expression of *kpsMT* in periodontal strains (35%) was lower than that described in uropathogenic strains of *E. coli* (61.3%) (Paniagua-Contreras et al., 2017) and that described in cervico-vaginal strains of *E. coli* (72.5%) (Monroy-Pérez et al., 2020); however, the expression of *ompT* (28%) in our periodontal strains was higher than that described in uropathogenic strains of *E. coli* (9.8%) (Paniagua-Contreras et al., 2017). Many of the virulence genes identified in these *E. coli* strains are frequently transferred horizontally through pathogenicity islands (PAIs) (Zakaria et al., 2022), which would explain the high frequency of expression of the *malX* gene (45/100) detected among our periodontal strains.

In the research previously carried out by our work group (Hernández-Jaimes et al., 2022) we described that the genes *tsh* (3/100), *iuc* (20/100), *focG* (9/100), *iss* (8/100), and *bmaE* (0/100) were detected at low frequency in the *E. coli* strains, and in this new study they were not expressed. This result suggests that the culture experimental conditions might influence the expression of these genes in some specific strains. Therefore, niche-specific culture conditions could provide more precise insights. Another possibility is that the expression of these genes was not necessary for infection of the A549 cell line. This is one of the limitations of this work. Future studies could focus on using panels of gingival epithelial cell lines to provide a more accurate representation of the periodontal niche. Furthermore, exploring different time points and environmental conditions could offer deeper insights into the dynamics of virulence gene expression (Domka et al., 2007). Another experimental limitation is that virulence expression can be influenced by the bacterial communities of the biofilm formation in the oral cavity, as has been shown in experimental models (Sbordone and Bortolaia, 2003; Naves et al., 2008). These factors can be evaluated in forthcoming studies.

Regarding the digree of periodontal disease, the frequency of detection of the antibiotic and disinfectant resistance genes showed no statistically significant differences (**Table 2**). However, the genes *aadA1, aac(3)-IV, sul-2, sul-3, bla_TEM_, bla_oxa-1-like_*, *cat1, dfrA1*, and *marR-marO* were detected more frequently in strains from patients with gingivitis, while *sul-1, bla_SHV_, bla_CITM_, tet(A), tet(B), qnr*, and *flor* were detected more prevalent in strains from patients with chronic periodontitis, and *qacEA1* was detected more frequently in strains from patients with moderate periodontitis. The high frequency of detection of the antibiotic resistance genotype detected in *E. coli* strains associated with periodontal disease coincides with the resistance phenotype for beta-lactams (ampicillin, carbenicillin, cephalothin and cefotaxime), quinolones (norfloxacin and ciprofloxacin), nitrofurantoin, aminoglycosides (amikacin and netilmicin), and trimethoprim-sulfamethoxazole, as described in our previous study on the same *E. coli* strains (Hernández-Jaimes et al., 2022). Another study on aerobic periodontal strains, including *E. coli*, reported high percentages of resistance to beta-lactams (cephazolin, cephamezin and cephdazidim), tetracycline, and quinolones (ciprofloxacin) (Nemsadze et al., 2006). The high frequency of detection of sulfonamide, beta-lactam, tetracycline, MAR locus, and disinfectant resistance genes among the strains reflects the notable increase in multidrug-resistance in *E. coli* strains associated with periodontal disease, which could make medical treatment of infections difficult. Studies on multidrug-resistance in periodontal strains are scarce; however, in a large review study on multidrug-resistance in Mexico in uropathogenic strains (UPEC) during a period from 2007 to 2022, high percentages of resistance were described for penicillin, carbenicillin, tetracycline, trimethoprim/sulfamethoxazole, pefloxacin, nalidixic acid, levofloxacin, norfloxacin, cephalothin, cefotaxime, and ciprofloxacin (Ballesteros-Monrreal et al., 2023).

We identified a broad correlation between the expression percentages of adhesion genes (*fimH* and *iha*), iron uptake systems (*irp-2*, *feoB* and *fyuA*), and protectins (*traT*) with the frequency of detection of the genes for sulfonamide (*sul-2*), beta-lactams (*bla_TEM_*), tetracycline (*tet(A*)), the MAR locus (*marR-marO*), and the frequency of detection of the gene encoding resistance to disinfectants (*qacEA1*) in gingivitis strains (p < 0.05; **Table 3**). Similarly, in strains obtained from patients with moderate periodontitis (**Table 4**), a higher correlation was found between the expression of adhesion genes (*fimH*) and iron uptake systems (*irp-2, feoB* and *fyuA*) and frequency of detection of the genes encoding resistance to beta-lactam (*bla_SHV_* and *bla_TEM_*), the MAR locus (*marR-marO*), and resistance to disinfectants (*qacEA1*), while in strains obtained from patients with chronic periodontitis (**Table 5**), the correlation between the highest expression of virulence genes was observed for adhesion genes (*fimH* and *iha*), iron uptake genes (*irp-2, feoB* and *fyuA*), protectins (*traT*) and pathogenicity islands (*malX*) whit the frequency of detection of the beta-lactam genes (*bla_SHV_*), the MAR locus (*marR-marO*), and the gene encoding resistance to disinfectants (*qacEA1*). These results are clinical relevance and show that during *in vitro* infection of the A549 line, there was a broad collective participation in the expression of virulence genes correlated with the detection of the multidrug-resistance genotype and disinfectants, which could contribute to the acuteness or chronicity of periodontal infections; therefore, it is necessary to improve the prevention and medical treatment schemes against periodontal infections associated with *E. coli* in Mexico.

Pulsed field gel electrophoresis (PFGE) analysis revealed a wide array of different PFtypes distributed in two major clades (A and B; **Figure 1**). Notably, no single PFtype emerged as dominant across all strains, with each strain exhibiting expression patterns sapannig from two to 27 genes. The diverse distribution of expression profiles of genes involved in adhesion, tissue damage, and degradation of host defenses, nutrition, and immune response evasion related to PFtypes and to the genotype associated with resistance to antibiotics (streptomycin, gentamicin, sulfonamide, beta-lactams, chloramphenicol, tetracycline, trimethoprim, quinolones, the Mar locus) and disinfectants, evinces the high pathogenic capacity of these strains and its potential to cause chronic infections.

Unsupervised hierarchical clustering analysis demonstrated that *E. coli* strains from patients with gingivitis, P. moderate and P. Chronic strains were distributed into two large groups according to the broad composition of virulome expression related to the frequency of the genotype of resistance to antibiotics and disinfectants, so these strains may contribute significantly to the severity and cronicity of periodontal infections.

## 5 Conclusions

This is the first study conducted in Mexico to globally analyze the expression of the virulome related to antimicrobial and disinfectant resistance genotypes and PFtype in periodontal strains of *E. coli*. These findings emphasize the high pathogenic capacity of *E. coli* as an emerging bacterium associated with periodontal diseases.

## Data Availability

The original contributions presented in the study are included in the article/supplementary material. Further inquiries can be directed to the corresponding authors.
